# Investigating the Sensitivity of Nasal or Throat Swabs: Combination of Both Swabs Increases the Sensitivity of SARS-CoV-2 Rapid Antigen Tests

**DOI:** 10.1128/spectrum.00217-22

**Published:** 2022-06-28

**Authors:** Barbara L. Goodall, Jason J. LeBlanc, Todd F. Hatchette, Lisa Barrett, Glenn Patriquin

**Affiliations:** a Department of Medicine, Dalhousie University, Halifax, Nova Scotia, Canada; b Department of Pathology, Dalhousie University, Halifax, Nova Scotia, Canada; c Division of Microbiology, Department of Pathology and Laboratory Medicine, Nova Scotia Health, Halifax, Nova Scotia, Canada; d Department of Microbiology and Immunology, Dalhousie University, Halifax, Nova Scotia, Canada; Labcorp

**Keywords:** COVID-19, SARS-CoV-2, rapid, antigen, nasal, throat

## Abstract

The COVID-19 pandemic has been hallmarked by several waves of variants of concern (VoCs), each with novel challenges. Currently, the highly transmissible Omicron VoC is predominant worldwide, and sore throat is common, among other cold-like symptoms. Anecdotes on social media have suggested that sampling one’s throat can increase the sensitivity for Omicron detection by antigen-based rapid testing devices (Ag-RDTs). This work aimed to improve the local testing strategy and determine whether the sensitivity of Ag-RDTs designed for nasal sampling is altered with the use of self-administered throat swabs in self-perceived asymptomatic individuals. This investigation used a common Ag-RDT (i.e., Abbott Panbio COVID-19 Ag rapid test device) to compare three sampling sites: nasal swab, throat swab, and combined nasal/throat. All Ag-RDT results were confirmed with molecular testing from residual test buffer. Compared to reverse transcriptase PCR (RT-PCR), samples from nasal or throat swabs each detected 64.5% of SARS-CoV-2 cases; however, combining the contributions of each swab increased the positive percent agreement (PPA) with RT-PCR to 88.7%. This trend was also evident with the Rapid Response Ag-RDT (BTNX), which uses more flexible swabs than does the Panbio. When nasal swab collection was compared to paired sampling of the nose/throat using a single swab with the Panbio Ag-RDT, the PPAs were 68.4% and 81.6%, respectively. No false-positive results were observed with nasal, throat, or combined nasal/throat sampling. Self-administered throat and nasal/throat swabs both had >90% acceptability. These findings support the use of self-collected combined nasal/throat sampling for Ag-RDT-based SARS-CoV-2 detection in self-perceived asymptomatic individuals.

**IMPORTANCE** This quality project demonstrates that combining the results of nasal and throat swabs or using a combined single swab of the throat and nares resulted in increased detection of SARS-CoV-2 using a rapid antigen test, in an asymptomatic population. Importantly, no false positives were detected, and over 90% of people were willing to perform the combination swab. These types of projects are instrumental in informing local practices to improve testing strategies. These data support the option of using a combined nasal/throat swab in our local setting to enhance the detection of Omicron.

## INTRODUCTION

The COVID-19 pandemic is an ongoing threat to global public health. Since the first cases were reported in December 2019, several SARS-CoV-2 variants of concern (VoCs) have emerged, causing multiple waves of infection globally (1). Recently the predominant VoC in many jurisdictions has been SARS-CoV-2 lineage B.1.1.529, designated Omicron by the World Health Organization (WHO) classification working group (1); this VoC is highly mutated, with over 50 mutations compared to ancestral reference genomes (2, 3). Most mutations are present in the gene encoding spike, the protein that is targeted by all current vaccines and plays a role in host receptor interactions to facilitate viral entry and subsequent replication. Among other Omicron mutations, four are in the gene encoding nucleocapsid, the target of most antigen-based rapid diagnostic tests (Ag-RDTs). While some recent data have alleviated initial concerns over the possibility of decreased sensitivity of Ag-RDTs with Omicron, there are other noted differences from previous VoCs (4). Omicron has increased transmission and reduced vaccine effectiveness (5, 6), and recent studies have suggested that there could be differences in tissue tropism (3, 7, 8).

Clinically prominent cold-like symptoms, especially marked sore throat (9), combined with reports of perceived decreased sensitivity of self-performed Ag-RDTs with bilateral nasal swab collection, have led to anecdotal recommendations to self-swab one’s throat (with or without nasal swabbing), contradicting manufacturer directions. These views have been widely propagated on social media and discussed and debated in recent print and televised media (10–12). From a virus perspective, the viral tropism and kinetics of Omicron may differ from previous variants (7, 8), adding some credence to the idea that virus may be in different parts of the respiratory system at different times.

Many factors can affect Ag-RDT sensitivity, including the timing and quality of specimen collection, the specimen type, and the testing method itself (13, 14). Previous work has also shown that deviating from the manufacturer’s instructions for rapid antigen tests can introduce errors that may yield inaccurate results (15). Misunderstanding erroneous test results can fuel misinformation and disinformation, ultimately undermining confidence in public health efforts and trust in health care professionals.

At a low-barrier volunteer-led community testing center (16), where samples were self-collected with coaching, this work sought to investigate whether Omicron could be detected using Ag-RDTs in asymptomatic individuals, to evaluate the potential benefits of throat sampling, and to assess the applicability of throat sampling to other Ag-RDTs using different swabs.

## RESULTS

### Comparison of nasal and throat samples using the Panbio Ag-RDT.

**FIG 1:**
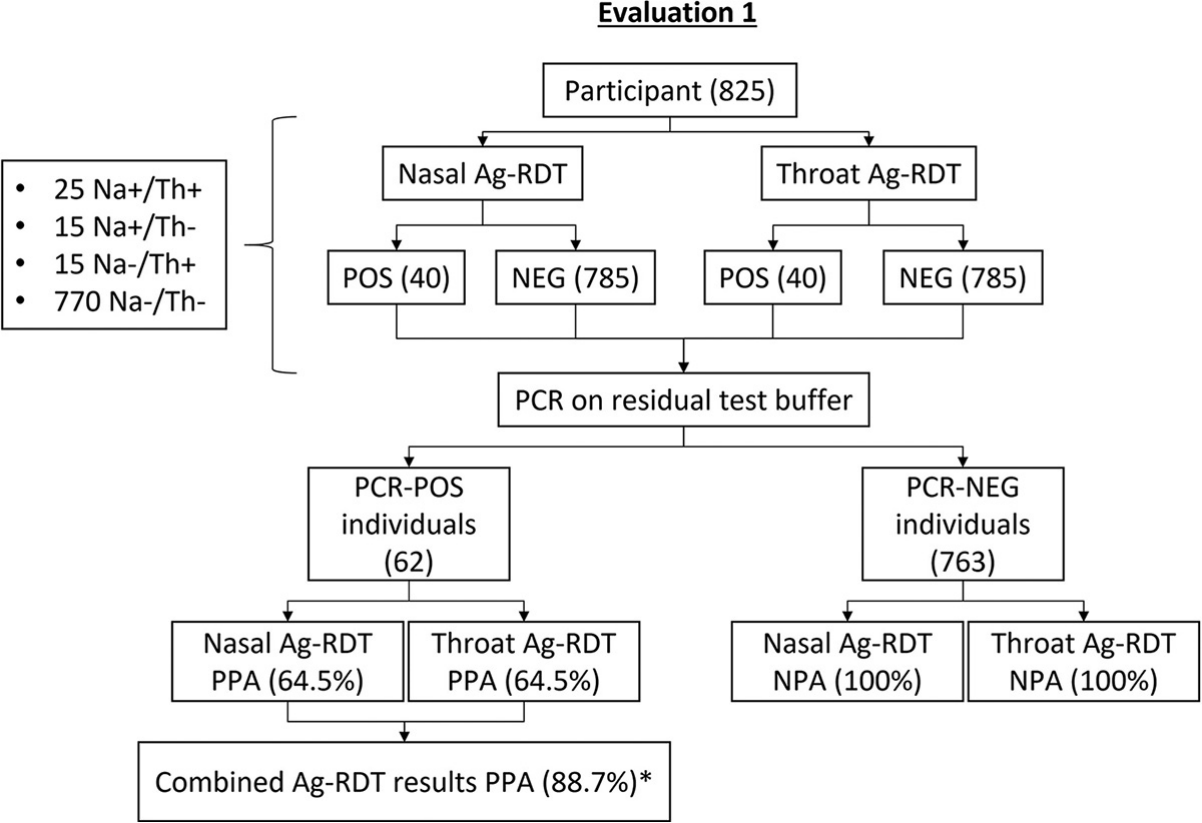
Recruitment and results of evaluation 1. Na, nasal; Th, throat; POS, positive; NEG, negative; Ag-RDT, antigen-based rapid diagnostic test; PPA, percent positive agreement; NPA, negative percent agreement; *significant *P* value, <0.05.

A total of 1,568 people attended the testing site during the 7-day investigation period, of whom 1,472 (93.9%) consented to participate. Of those who participated in evaluation 1, the phase of separate bilateral nasal and throat swabbing, 40 positive Panbio Ag-RDT results each were obtained with both the nasal and throat swabs, out of 62 reverse transcriptase PCR (RT-PCR)-positive individuals. As such, the positive percent agreement (PPA) for nasal or throat swabs was identical at 64.5% (95% confidence interval [CI], 52.1 to 75.3%) (Fig. 1). When detections attributed to either swab were combined in the same analysis, 55 of 62 individuals were identified as positive using the Panbio Ag-RDT, for a combined PPA of 88.7% (95% CI, 78.2 to 94.7%) (Fig. 1). None of the false-negative Ag-RDTs from either anatomical site had *C_T_* values of <29. No false positives were observed, and as such, the negative percent agreement (NPA) of each swab was 100% (95% CI, 99.5 to 100.0%). Of note, all RT-PCR-positive samples featured an S-gene target dropout (see Table S1 in the supplemental material), which is consistent with the circulating Omicron VoC. Similarly increased detection of SARS-CoV-2 was seen in a smaller group of Panbio-positive participants after adding the results from nasal and throat swabs with the BTNX Rapid Response Ag-RDT (Table S2).

### Evaluation of a combined nasal/throat swab using Panbio.

**FIG 2:**
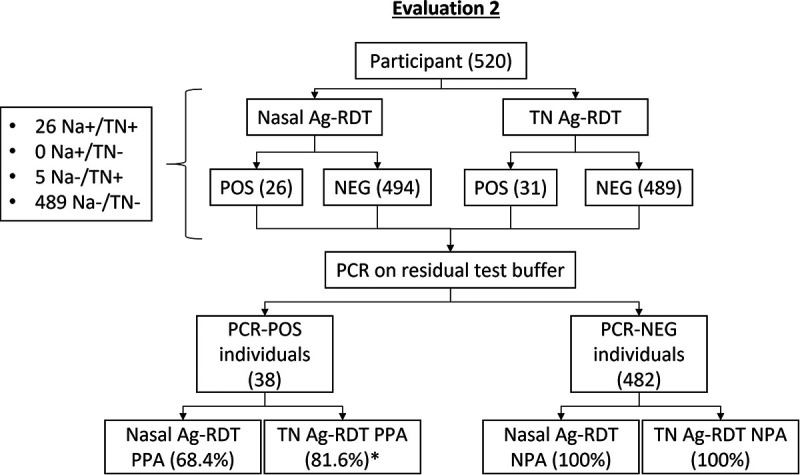
Recruitment and results of evaluation 2. Na, nasal; TN, throat/nasal; POS, positive; NEG, negative; Ag-RDT, antigen-based rapid diagnostic test; PPA, percent positive agreement; NPA, negative percent agreement; *significant *P* value, <0.05.

After confirming the PPAs of throat and nasal swabs alone, the performance characteristics of a combined nasal/throat swab was evaluated to facilitate the practical applications of Ag-RDT sampling of both anatomical sites. For this phase of the quality project (evaluation 2), participants were asked to provide a combined nasal/throat swab after having completed the standard bilateral nasal swab, and each swab was subjected to the Panbio Ag-RDT. A total of 520 individuals participated, of whom 38 were PCR positive by either swabbing method (Fig. 2). Bilateral nasal swabs resulted in 26 positive Ag-RDTs, with a PPA of 68.4% (95% CI, 51.4 to 82.5%). In comparison, combined nasal/throat swabbing identified 31 positive participants, for a PPA of 81.6% (95% CI, 65.7 to 92.3%). None of the false-negative Ag-RDT results from either anatomical site had *C_T_* values of <30 (Table S3). As no false positives were detected in the evaluation period, an NPA of 100% (95% CI, 99.2 to 100.0%) was obtained for both swabs.

## DISCUSSION

A global crisis such as a pandemic and the rapid dissemination of information through social media can fuel the perpetuation of anecdotes, misinformation, and disinformation, which can hamper public health efforts and trust in health care professionals. This quality project assessed assertions from media (social, print, televised) urging the public to swab their throats in addition to (or instead of) their nares for testing using Ag-RDTs. In this project, a combined collection of both nasal and throat samples increased Ag-RDT detection compared to the manufacturer-recommended sampling of bilateral nares alone.

Given the frequency of cold-like symptoms and complaints of sore throat during this period of high prevalence of Omicron, there was likely some validity to the media accounts of false-negative nares swabs, with subsequent follow-up positive throat swabs. Many of the Ag-RDTs available to the public have been approved for use with nasal swabs and are not approved for sampling saliva or the throat; previous work shows the potential for invalid results when using an assay outside the manufacturer’s instructions (15). Without careful evaluation of this widely publicized off-label use, variables such as the test timing (and the time between nasal and throat tests), testing kit manufacturer, swab types, and buffers cannot be controlled. Importantly, confirmation of test results with more sensitive and specific assays (such as RT-PCR) cannot be easily performed to corroborate individual anecdotes. Investigations in this work allowed for careful evaluation of SARS-CoV-2 detection using Ag-RDTs with nasal and throat swabs.

Prior to undertaking this evaluation, one of the primary concerns for the wide use of throat-based collection for Ag-RDT testing was the potential for false positives. While food and beverages could potentially impact the performance of Ag-RDTs or molecular testing (17), all of the Ag-RDT-positive throat samples in this project were confirmed positive by RT-PCR. Based on our experience with community-based testing over the last year, the estimated false-positive rate was between 2 and 4 per 1,000 individuals, which is consistent with manufacturer claims. Given the widespread activity of Omicron in the community, it was not surprising that few false positives were observed.

Apart from ensuring Ag-RDT specificity with the use of a throat-based collection, understanding its potential impacts on sensitivity is also crucial. The PPA with RT-PCR of the Ag-RDT for Omicron detection in this investigation was 82% (versus RT-PCR) when using the combined nasal and throat swabs, which is on the higher end of estimates previously reported for both Omicron and non-Omicron variants. The most recent Cochrane review of the performance of Ag-RDTs prior to the Omicron wave suggested that the sensitivity of the Panbio assay was between 50 and 92% (18). Although the U.S. Food and Drug Administration (FDA) recently issued a statement that antigen tests may have reduced sensitivity for the detection of Omicron (19), real-world data were lacking. Consistent with others (4, 20), the National Microbiology Laboratory (NML) in Canada recently assessed the performance of several Ag-RDTs for Omicron detection, and the results for Panbio and BTNX were comparable to previous evaluations using other SARS-CoV-2 lineages (NML, unpublished communication).

The added detection from sampling the throat could be due to several factors. A sampling error or less vigorous sampling of one site versus the other would influence the quality of the specimen. With the use of residual test buffer (RTB) following Ag-RDT testing, molecular testing could be performed to understand the SARS-CoV-2 cases that would be potentially missed by Ag-RDTs using nasal and throat swab-based collections. As expected for any Ag-RDT, there were false-negative Ag-RDT results detected by RT-PCR, but these were equally distributed between the nasal and throat-based collections. It is also possible that the discrepancy between nasal and throat results is attributable to preferential replication of the Omicron variant in these anatomical sites at different time points in infection. Prior to the first descriptions of Omicron, reports of RT-PCR from comparisons of nasopharyngeal and oropharyngeal/nares (21) and nasal and oropharyngeal (22) swabs showed little difference. On the other hand, in symptomatic nonhospitalized patients in South Africa, saliva samples detected Omicron more often than midturbinate swabs (100 versus 86%, respectively) (8), which was opposite from the performance reported for previously circulating variants. Similarly, in a preprint non-peer reviewed publication of a small subset of individuals in a high-risk occupational case cohort, the viral load in saliva specimens peaked 1 to 2 days prior to the peak in those observed with nasal swab collections (23). These early data suggest that there may be a different tissue tropism for the Omicron variant compared to prior circulating lineages of SARS-CoV-2 (7).

Most individuals being tested agreed to participate in this project, with only approximately 6% refusing, indicating that a self-administered throat swab is an acceptable method for COVID-19 testing. The tolerability of throat swabs and of combined nasal/throat swabs appeared to be high, without voiced concerns for a swab preference. Participants often performed the self-administered throat swab correctly with minimal coaching. Many participants experienced a gag reflex during the self-administered throat swab, and one individual vomited. This in-person volunteer observation and coaching could easily be translated to a brief instructional video for at-home/occupational use.

This investigation is not without limitations. Our adult population is from a single center during a 7-day period, at a community testing center. There was no collection of clinical information related to patient demographics, timing from exposure, immunization history, or recent consumption of food or drink. One of the criteria for attending the testing site was self-described asymptomatic status. However, mild symptoms may not have been recognized as Omicron infection. For those with recognized symptoms, it is possible that the sensitivity and specificity may differ from those determined in this project. From a practical standpoint, determination of symptom onset would likely not be a requisite for determining the swabbing technique. This is particularly important as self-directed testing and management become a tool of endemic living; these data add confidence to the ability of individuals to adequately perform reliable sample collection in a real-world setting.

Although this project focused on the Abbott Panbio Ag-RDT, the concordant results obtained using the BTNX Rapid Response Ag-RDT suggest that this enhanced detection rate using a combined swab is applicable to other lateral flow tests, although further studies are warranted. While molecular confirmation with RT-PCR was available, RTB is not a typical testing matrix. Despite our previous validations of RTB for this application (16), this approach is not as sensitive as an independent collection of swabs for RT-PCR testing using a dedicated transport medium. As such, the PPA of Ag-RDTs may be overestimated in this project, but this would apply to each swab type. However, not all real-world settings with vaccinated individuals still aim to identify the presence of molecular viral genetic material. The use of RT-PCR in this case was intended to provide a traditional comparison for laboratory testing purposes (24), which may not be relevant in a postvaccine pragmatic setting (25). It should be noted that the use of RTB for RT-PCR testing avoids the additional nasal and throat collections for Ag-RDT and molecular testing. This work showed that both the throat swab alone and the combined nasal/throat swab were well accepted by participants; however, it is unclear whether the same level of participation or acceptability would occur if 4 swabs were required for evaluations (2 each for Ag-RDT and RT-PCR testing). Finally, as Omicron was the only SARS-CoV-2 lineage circulating at the time, whole-genome sequencing was not performed on the nucleic acids extracted from each swab; however, all positive samples lacked amplification of the S-gene target, which is consistent with, but not confirmatory of, detection of Omicron (26). This may be relevant as different variants may be optimally detected with sampling of different anatomical sites, and our results are only generalizable to this presumed Omicron variant with potential tropism differences compared to previously circulating SARS-CoV-2 lineages.

Overall, this work provides early evidence that combined throat and bilateral nasal swabs maximize the ability of Ag-RDTs to identify the Omicron variant. These types of projects are instrumental in testing anecdotal reports advocating the off-label use of approved testing methods to inform the local community practice and increase confidence for those making swabbing recommendations to various stakeholders and users, including asymptomatic people performing self-Ag-RDTs.

## MATERIALS AND METHODS

### Sample collection and SARS-CoV-2 Ag-RDTs.

**FIG 3:**
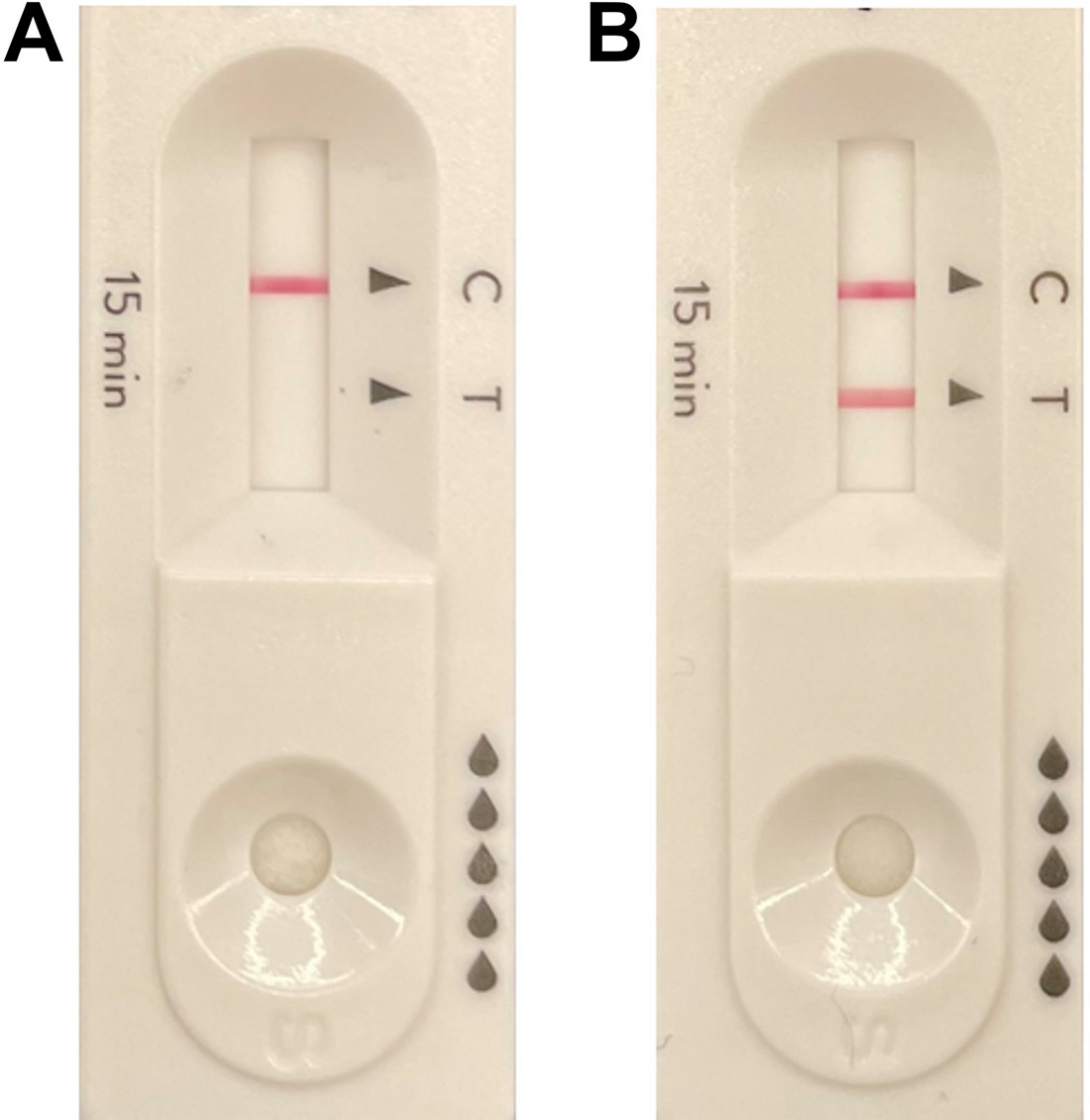
Representative sample of a single participant’s results using the Abbott Panbio antigen-based rapid testing device for (A) nasal sampling alone and (B) combined nasal/throat sampling. Bands represent the control line (C) and test line (T), indicating a negative result in panel A and the detection of SARS-CoV-2 in panel B.

The participants were self-referred community members attending an urban rapid testing location available specifically for those who self-identified as being asymptomatic (16), over a 7-day period in January 2022. The individuals’ reasons for testing were not known, no medical records were accessed, and no clinical assessments were performed. For evaluation 1, after providing verbal consent, participants were verbally instructed on, and observed performing, a self-collected bilateral nasal swab (by inserting the swab approximately 2 cm into the nostril and rotating five times per nostril), in keeping with the manufacturer’s instructions. In addition, the participants were then guided on self-collection of a posterior oropharyngeal swab, with the aid of an anatomical diagram of the mouth (rubbing the swab five times on the back of the throat), which is a method not endorsed by the manufacturer. In evaluation 2, new participants were instructed on self-swabbing for a bilateral nasal sample, followed by a combined throat/bilateral nasal sample (not included in the manufacturer’s instructions). All swabs were collected by the participants with minimal coaching by trained volunteers using the Panbio COVID-19 Ag rapid test device (Abbott Rapid Diagnostics, Jena, Germany). The swabs included with other Ag-RDTs differ in wand flexibility and may have different characteristics for throat sampling. Therefore, for a subset of participants with a positive Panbio Ag-RDT (from either anatomical site), individual self-collected swabs from the anterior nares bilaterally (according to the manufacturer’s instructions) and throat (not endorsed by the manufacturer) were repeated and processed using the Rapid Response COVID-19 antigen rapid test device (BTNX Inc., Markham, ON). All Ag-RDTs were interpreted by trained volunteer testing site staff according to the manufacturer’s instructions, with a visualized test (T) band considered positive (in conjunction with a visualized control [C] band), regardless of intensity (Fig. 3), as is directed by the package insert.

### SARS-CoV-2 RT-PCR.

Real-time RT-PCR was performed on residual test buffer (RTB) from the Ag-RDT kits to confirm positive antigen detections and to investigate the possibility of false-negative results. Though the sample type (RTB) and methods for nucleic acid amplification testing (NAAT) are not accepted or endorsed by the instrument and kit manufacturers, the method has been validated previously (16). Briefly, the remaining fluid in the Ag-RDT tubes along with the used swabs for all participants (Ag-RDT positive or negative) were transported to a central laboratory, and 200 μL viral transport medium (VTM) (Rodoxica, Little Rock, AR) was added to each tube. Following gentle vortexing for 5 s, the VTM/fluid was transferred to a microcentrifuge tube, and 150 μL was subjected to a total nucleic acid (TNA) extraction on a MagNA Pure LC 2.0 instrument (Roche Diagnostics Ltd., Roltkreuz, Switzerland), according to the manufacturer’s instructions. TNAs were eluted in a volume of 50 μL, and 5 μL was used as the template in a real-time RT-PCR using the TaqPath COVID-19 Combo kit (Life Technologies Corp., Frederick, MD) on an Applied Biosystems thermocycler, according to the manufacturer’s directions.

### Data analysis.

The results were defined at the level of the participant, using RT-PCR as the reference method. Any antigen-positive result from any individual’s swabs confirmed by RT-PCR was considered positive. A positive Ag-RDT result that failed to confirm with RT-PCR testing was interpreted as a false positive. False-negative Ag-RDT results were defined by the detection of SARS-CoV-2 by RT-PCR in the absence of an Ag-RDT positive result. Concordant negative results by both Ag-RDT and RT-PCR were considered negative. Descriptive statistics were used to report participation and testing outcomes, using MedCalc where appropriate. Positive percent agreement (PPA) and negative percent agreement (NPA) with RT-PCR were reported with 95% confidence intervals (CI) for all investigations, with the exception of specificity for BTNX, which was not calculated due to the lack of appropriate denominator. *P* values were determined using McNemar’s test with Yate’s correction, using https://www.omnicalculator.com/statistics.

### Ethics.

The primary goal of this project was to improve virus detection in the provincial testing strategy, and it was therefore deemed a quality initiative, exempt from review by the Nova Scotia Health Research Ethics Board (submission number 1027644). The specimens tested were obtained from consenting participants, and all data related were provided anonymized, deidentified, and used solely with the intent to evaluate the performance characteristics of the different swab types for rapid antigen testing programs used in Nova Scotia.
