# Supplementary material for: Investigating the Sensitivity of Nasal or Throat Swabs: Combination of Both Swabs Increases the Sensitivity of SARS-CoV-2 Rapid Antigen Tests
Source: Microbiol Spectr. 2022 Jun 28;10(4):e00217-22. doi: 10.1128/spectrum.00217-22 (PMC9430836; doi:10.1128/spectrum.00217-22)
Supplement: Supplemental file 1 — Supplemental material. Download spectrum.00217-22-s0001.pdf, PDF file, 0.3 MB [file spectrum.00217-22-s0001.pdf]

Table S1. Nasal vs. Throat (Panbio)

Participant number	Nasal						Throat				
	Panbio	Taqpath					Panbio	Taqpath			
	Result	N	Orf1a	S	Result		Result	N	Orf1a	S	Result
PB1	POS	29.48	29.13	NEG	POS		NEG	35.13	35.07	NEG	POS
PB2	NEG	34.17	33.17	NEG	POS		POS	30.19	30.03	NEG	POS
PB3	POS	31.68	30.66	NEG	POS		POS	29.18	28.83	NEG	POS
PB4	NEG	35.18	35.23	NEG	POS		POS	31.01	30.78	NEG	POS
PB5	POS	21.32	20.2	NEG	POS		NEG	33.69	31.31	NEG	POS
PB6	POS	30.63	29.59	NEG	POS		NEG	35.41	34.57	NEG	POS
PB7	POS	30.28	30.64	NEG	POS		POS	28.54	28.38	NEG	POS
PB8	NEG	33.34	33.86	NEG	POS		POS	29.35	29.78	NEG	POS
PB9	NEG	34.82	34.06	NEG	POS		POS	33.56	34.06	NEG	POS
PB10	POS	29.35	29.78	NEG	POS		POS	27.54	27.49	NEG	POS
PB11	POS	20.34	19.87	NEG	POS		POS	18.98	19.17	NEG	POS
PB12	POS	30.08	29.74	NEG	POS		NEG	33.48	33.22	NEG	POS
PB13	POS	27.09	26.61	NEG	POS		POS	27.69	27.44	NEG	POS
PB14	POS	28.94	28.17	NEG	POS		POS	31.98	31.16	NEG	POS
PB15	POS	30.28	30.03	NEG	POS		NEG	36.68	NEG	NEG	POS
PB16	NEG	34.12	33.68	NEG	POS		POS	29.32	32.33	NEG	POS
PB17	POS	28.87	29.01	NEG	POS		NEG	29.62	29.41	NEG	POS
PB18	POS	23.92	23.66	NEG	POS		POS	25.21	25.43	NEG	POS
PB19	NEG	37.84	37.04	NEG	POS		POS	29.6	30.67	NEG	POS
PB20	NEG	36.18	36.08	NEG	POS		POS	30.64	30.94	NEG	POS
PB21	POS	31.66	31.61	NEG	POS		POS	31.23	33.85	NEG	POS
PB22	POS	26.01	25.86	NEG	POS		POS	32.67	32.18	NEG	POS
PB23	POS	30.33	30.14	NEG	POS		NEG	31.31	30.49	NEG	POS
PB24	POS	32.21	33.25	NEG	POS		POS	27.61	27.16	NEG	POS
PB25	POS	30.03	30.21	NEG	POS		POS	33.54	33.63	NEG	POS
PB26	NEG	31.61	30.5	NEG	POS		POS	31.04	30.21	NEG	POS
PB27	POS	32.83	32.91	NEG	POS		POS	28.80	28.65	NEG	POS
PB28	POS	26.99	26.49	NEG	POS		POS	28.12	28.13	NEG	POS
PB29	NEG	35.46	38.94	NEG	POS		POS	29.05	30.09	NEG	POS
PB30	POS	25.41	25.51	NEG	POS		POS	33.12	33.02	NEG	POS
PB31	POS	27.48	28.72	NEG	POS		NEG	33.41	34.42	NEG	POS
PB32	NEG	33.36	34.07	NEG	POS		POS	31.30	32.04	NEG	POS
PB33	NEG	33.92	36.32	NEG	POS		POS	31.23	33.09	NEG	POS
PB34	NEG	33.36	34.07	NEG	POS		POS	31.3	32.04	NEG	POS
PB35	POS	28.38	29.15	NEG	POS		NEG	33.19	34.33	NEG	POS
PB36	POS	24.14	25.63	NEG	POS		POS	25.26	25.59	NEG	POS
PB37	POS	32.43	33.10	NEG	POS		NEG	33.45	34.90	NEG	POS
PB38	POS	28.02	29.11	NEG	POS		NEG	31.92	32.98	NEG	POS
PB39	NEG	31.63	33.01	NEG	POS		POS	27.8	28.97	NEG	POS
PB40	POS	31.81	32.32	NEG	POS		NEG	31.12	31.98	NEG	POS
PB41	POS	23.23	23.66	NEG	POS		NEG	32.47	33.15	NEG	POS
PB42	POS	23.70	24.65	NEG	POS		POS	31.17	32.56	NEG	POS
PB43	POS	28.14	29.42	NEG	POS		POS	31.08	35.53	NEG	POS
PB44	POS	28.79	29.83	NEG	POS		NEG	32.41	33.02	NEG	POS

PB45	POS	31.57	33.62	NEG	POS		POS	31.12	32.23	NEG	POS
PB46	POS	28.77	29.13	NEG	POS		POS	28.43	29.17	NEG	POS
PB47	POS	20.23	20.65	NEG	POS		POS	30.09	32.64	NEG	POS
PB48	POS	26.50	27.07	NEG	POS		POS	21.39	21.78	NEG	POS
PB49	POS	27.16	27.36	NEG	POS		POS	27.15	27.83	NEG	POS
PB50	POS	31.11	31.49	NEG	POS		NEG	33.41	34.87	NEG	POS
PB51	POS	26.12	26.26	NEG	POS		POS	31.1	33.17	NEG	POS
PB52	NEG	32.82	33.01	NEG	POS		POS	29.73	30.13	NEG	POS
PB53	POS	27.01	27.92	NEG	POS		POS	27.66	28.64	NEG	POS
PB54	NEG	32.17	33.15	NEG	POS		POS	30.83	31.15	NEG	POS
PB55	POS	27.96	28.49	NEG	POS		POS	31.8	32.78	NEG	POS
PB56	NEG	30.61	31.07	NEG	POS		NEG	36.68	37.03	NEG	POS
PB57	NEG	32.80	33.44	NEG	POS		NEG	34.14	36.16	NEG	POS
PB58	NEG	34.99	36.4	NEG	POS		NEG	34.12	36.72	NEG	POS
PB59	NEG	NEG	NEG	NEG	NEG		NEG	30.45	30.7	NEG	POS
PB60	NEG	NEG	NEG	NEG	NEG		NEG	29.54	30.24	NEG	POS
PB61	NEG	NEG	NEG	NEG	NEG		NEG	NEG	35.56	NEG	POS
PB62	NEG	NEG	NEG	NEG	NEG		NEG	35.86	NEG	NEG	POS
Negatives (n=763)	NEG	NEG	NEG	NEG	NEG		NEG	NEG	NEG	NEG	NEG

Table S2. Nasal vs. Throat (BTNX)

Participant number	Nasal						Throat				
	BTNX	Taqpath					BTNX	Taqpath			
	Result	N	Orf1a	S	Result		Result	N	Orf1a	S	Result
BTNX1	NEG	32.08	33.11	NEG	POS		POS	26.49	27.02	NEG	POS
BTNX2	NEG	33.41	34.14	NEG	POS		POS	31.82	32.26	NEG	POS
BTNX3	POS	27.96	28.54	NEG	POS		POS	31.05	32.00	NEG	POS
BTNX4	POS	31.34	32.84	NEG	POS		NEG	27.71	28.56	NEG	POS
BTNX5	POS	29.20	29.11	NEG	POS		NEG	27.43	28.05	NEG	POS
BTNX6	POS	28.88	29.28	NEG	POS		NEG	33.00	34.12	NEG	POS
BTNX7	POS	28.96	30.36	NEG	POS		NEG	37.88	NEG	NEG	NEG
BTNX8	POS	31.68	33.17	NEG	POS		POS	32.83	34.06	NEG	POS
BTNX9	POS	26.62	27.07	NEG	POS		NEG	29.63	30.57	NEG	POS
BTNX10	POS	25.27	25.55	NEG	POS		POS	28.31	29.18	NEG	POS
BTNX11	POS	29.39	30.57	NEG	POS		POS	31.02	31.89	NEG	POS
BTNX12	POS	28.34	29.15	NEG	POS		POS	29.43	30.68	NEG	POS
BTNX13	NEG	32.80	33.76	NEG	POS		POS	31.29	31.13	NEG	POS
BTNX14	POS	29.18	29.69	NEG	POS		POS	28.13	28.63	NEG	POS
BTNX15	NEG	37.12	36.91	NEG	POS		POS	22.85	23.77	NEG	POS
BTNX16	POS	24.92	25.51	NEG	POS		POS	31.38	33.16	NEG	POS
BTNX17	POS	31.33	33.09	NEG	POS		POS	34.03	35.88	NEG	POS
BTNX18	NEG	30.87	30.91	NEG	POS		POS	27.81	28.12	NEG	POS
BTNX19	POS	32.30	32.47	NEG	POS		POS	21.49	21.73	NEG	POS
BTNX20	POS	32.45	31.87	NEG	POS		POS	28.94	29.63	NEG	POS
BTNX21	NEG	31.84	31.98	NEG	POS		POS	29.04	28.82	NEG	POS
BTNX22	POS	26.91	26.96	NEG	POS		POS	27.53	27.61	NEG	POS
BTNX23	POS	27.37	27.55	NEG	POS		NEG	31.04	31.77	NEG	POS
BTNX24	POS	32.85	32.54	NEG	POS		POS	28.3	28.96	NEG	POS
BTNX25	POS	28.82	29.41	NEG	POS		POS	26.16	27.39	NEG	POS
BTNX26	NEG	37.12	36.13	NEG	POS		NEG	36.87	37.10	NEG	POS
BTNX27	POS	26.90	27.08	NEG	POS		NEG	34.23	34.67	NEG	POS
BTNX28	POS	25.22	26.06	NEG	POS		NEG	29.87	30.37	NEG	POS
BTNX29	POS	24.49	24.19	NEG	POS		NEG	33.23	33.56	NEG	POS
BTNX30	POS	26.12	26.62	NEG	POS		POS	29.73	30.1	NEG	POS
BTNX31	POS	27.93	28.76	NEG	POS		POS	28.85	29.71	NEG	POS
BTNX32	POS	31.41	31.78	NEG	POS		NEG	36.89	35.97	NEG	POS
BTNX33	POS	33.13	34.20	NEG	POS		NEG	34.15	34.46	NEG	POS
BTNX34	POS	31.72	32.49	NEG	POS		POS	32.63	32.26	NEG	POS
BTNX35	POS	28.78	30.01	NEG	POS		POS	30.63	31.52	NEG	POS
BTNX36	POS	34.88	35.96	NEG	POS		NEG	NEG	NEG	NEG	NEG
BTNX37	POS	21.44	21.68	NEG	POS		POS	20.59	30.74	NEG	POS
BTNX38	POS	25.63	25.54	NEG	POS		POS	34.47	34.67	NEG	POS
BTNX39	POS	30.95	30.30	NEG	POS		NEG	NEG	NEG	NEG	NEG
BTNX40	NEG	33.13	33.66	NEG	POS		NEG	NEG	NEG	NEG	NEG
BTNX41	NEG	32.57	32.37	NEG	POS		POS	24.57	24.65	NEG	POS

Table S3. Nasal sample vs. Throat + Nasal combo swab (Panbio)

Participant number	Nasal						Nasal/throat				
	Panbio	Taqpath					Panbio	Taqpath			
	Result	N	Orf1a	S	Result		Result	N	Orf1a	S	Result
TN1	NEG	35.45	NEG	NEG	POS		POS	26.43	27.01	NEG	POS
TN2	POS	25.78	26.21	NEG	POS		POS	25.91	26.67	NEG	POS
TN3	POS	17.24	17.74	NEG	POS		POS	24.18	24.86	NEG	POS
TN4	POS	29.63	30.88	NEG	POS		POS	32.25	32.51	NEG	POS
TN5	NEG	33.12	33.69	NEG	POS		POS	27.67	28.07	NEG	POS
TN6	POS	18.91	19.77	NEG	POS		POS	25.36	25.89	NEG	POS
TN7	POS	32.35	34.02	NEG	POS		POS	29.88	30.21	NEG	POS
TN8	POS	24.12	24.65	NEG	POS		POS	20.64	21.63	NEG	POS
TN9	POS	29.81	30.08	NEG	POS		POS	29.62	29.97	NEG	POS
TN10	POS	34.32	34.71	NEG	POS		POS	33.82	NEG	NEG	POS
TN11	POS	33.87	34.27	NEG	POS		POS	34.16	35.64	NEG	POS
TN12	POS	31.12	30.94	NEG	POS		POS	28.28	28.32	NEG	POS
TN13	POS	21.83	21.68	NEG	POS		POS	24.52	24.82	NEG	POS
TN14	POS	19.75	19.71	NEG	POS		POS	19.42	19.37	NEG	POS
TN15	POS	31.11	31.08	NEG	POS		POS	31.58	31.79	NEG	POS
TN16	POS	30.76	31.20	NEG	POS		POS	29.35	29.70	NEG	POS
TN17	POS	26.98	26.87	NEG	POS		POS	28.79	28.89	NEG	POS
TN18	POS	24.28	24.33	NEG	POS		POS	22.70	22.64	NEG	POS
TN19	POS	31.87	31.78	NEG	POS		POS	31.03	31.11	NEG	POS
TN20	POS	30.57	30.73	NEG	POS		POS	30.24	30.62	NEG	POS
TN21	POS	25.63	25.95	NEG	POS		POS	25.16	25.09	NEG	POS
TN22	NEG	32.65	32.83	NEG	POS		POS	30.68	31.14	NEG	POS
TN23	NEG	NEG	NEG	NEG	NEG		POS	25.22	25.59	NEG	POS
TN24	POS	25.27	25.32	NEG	POS		POS	24.31	24.98	NEG	POS
TN25	POS	27.01	27.12	NEG	POS		POS	26.94	27.05	NEG	POS
TN26	POS	25.10	25.01	NEG	POS		POS	26.06	26.80	NEG	POS
TN27	POS	33.28	35.17	NEG	POS		POS	32.38	34.69	NEG	POS
TN28	POS	27.31	25.89	NEG	POS		POS	27.24	26.50	NEG	POS
TN29	POS	30.38	30.25	NEG	POS		POS	30.95	30.30	NEG	POS
TN30	POS	31.29	32.2	NEG	POS		POS	33.51	34.30	NEG	POS
TN31	NEG	34.25	35.25	NEG	POS		POS	24.77	24.68	NEG	POS
TN32	NEG	NEG	NEG	NEG	NEG		NEG	30.79	31.25	NEG	POS
TN33	NEG	35.77	NEG	NEG	POS		NEG	32.31	33.14	NEG	POS
TN34	NEG	34.51	NEG	NEG	POS		NEG	NEG	NEG	NEG	NEG
TN35	NEG	34.73	NEG	NEG	POS		NEG	NEG	NEG	NEG	NEG
TN36	NEG	NEG	NEG	NEG	NEG		NEG	35.44	35.83	NEG	POS
TN37	NEG	NEG	NEG	NEG	NEG		NEG	33.64	34.67	NEG	POS
TN38	NEG	35.22	35.02	NEG	POS		NEG	NEG	NEG	NEG	NEG
Negatives (n=482)	NEG	NEG	NEG	NEG	NEG		NEG	NEG	NEG	NEG	NEG
